# Ethnic Differences in Attending a Tertiary Dementia Clinic in Israel

**DOI:** 10.3389/fneur.2020.578068

**Published:** 2021-01-13

**Authors:** Polina Specktor, Rachel Ben Hayun, Natalia Yarovinsky, Tali Fisher, Judith Aharon Peretz

**Affiliations:** ^1^Cognitive Neurology Institute, Rambam Health Care Campus, Haifa, Israel; ^2^Technion – Israel Institute of Technology, Haifa, Israel

**Keywords:** dementia, cognitive, ethnicity, disparities, cohort, epidemiology, Arab, Jew

## Abstract

**Introduction:** Variations in lifestyle, socioeconomic status and general health likely account for differences in dementia disparities across racial groups. Our aim was to evaluate the characteristics of Arab (AS) and Jewish (JS) subjects attending a tertiary dementia clinic in Israel.

**Methods:** Retrospective data regarding subjects attending the Cognitive Neurology Institute at Rambam Health Care Campus between April 1, 2010, and April 31, 2016, for complaints of cognitive decline were collected from the institutional registry. AS and consecutive JS, aged ≥50 years without a previous history of structural brain disease, were included.

**Results:** The records of 6,175 visits were found; 3,246 subjects were ≥50 years at the initial visit. One hundred and ninety-nine AS and consecutive JS cases were reviewed. Mean age at first visit was 68.4 ± 8.8 for AS and 74.3 for JS (*p* < 0.0001). Mean education was 7.7 ± 4.8 years for AS and 11.3 years for JS (*p* < 0.0001). Mean duration of cognitive complaints prior to first visit did not differ between the groups. Initial complaints of both ethnicities were failing memory (97%) and behavioral changes (59%). Functional impairment was reported by 59% of AS and 45% of JS (*p* = 0.005). MMSE on first evaluation was 19.2 ± 7 for AS and 23.1 ± 5.9 for JS; *p* = 0.001. Alzheimer's disease was diagnosed in 32% AS and 23% JS, mild cognitive impairment in 12% AS and 21% JS. Normal cognition was diagnosed in 2% AS and 9% JS; *p* = 0.0001.

**Conclusions:** Compared to JS, AS attend a tertiary clinic when their cognitive impairment already affects their functional abilities providing a comprehensive benchmark for social health care interventions to reduce disparities.

## Introduction

The estimated number of people living with dementia worldwide currently is approaching 50 million. This number is estimated to triple by 2050, as a result of anticipated increase in middle and lower income countries due to aging of their populations (1). Ethnicity is one of the factors influencing dementia prevalence as it impacts health seeking behavior, stigma and perceived futility of the diagnosis (2). Various countries have reported dementia incidence and prevalence to be higher in ethnic minorities (3); alas, they use fewer medical services (4).

Few studies have evaluated the prevalence of dementia in the different ethnic groups in Israel. The largest ethnic groups in Israel are Israeli Jews followed by Israeli Arabs, further divided into Muslims, Christians and Druze, with a smaller number of other ethnicities. The prevalence of Alzheimer's disease (AD) in the 60 years and older Arab population in Israel is estimated as 10–20.5% (5, 6); this is four times higher than the estimated prevalence in the Jewish population (7, 8). Factors, such as high consanguinity rates and genetic pre-disposition (9, 10), high illiteracy rates (5) and lower socioeconomic status (11), have been considered to contribute to this disparity.

The present study sought to determine the usage of tertiary dementia clinic services in Israel among Arab (AS) and Jewish (JS) subjects, explore their characteristics and investigate their diagnosis and compliance with treatment. Exploring the reasons for the differences between these ethnic groups may help to direct service planning, health education and development of interventions.

## Materials and Methods

This is a retrospective study regarding subjects attending the Cognitive Neurology Institute at Rambam Health Care Campus (CNIR). It was reviewed and approved by the Institutional Review Boards of Rambam Health Care Campus (# 0680-19-RMB). Written informed consent from the participants was not required to participate in this study in accordance with national legislation and institutional requirements because the study involved medical record review with no subject contact.

The CNIR is a tertiary referral center for diagnosis and treatment of cognitive impairment and dementia, located in Haifa, north Israel. We collected retrospective data on subjects attending the CNIR for complaints regarding acquired cognitive decline between April 2010 and April 2016 from the computerized institutional registry. Ethnicity was determined according to subject's name, records of the ministry of internal affairs, self-report. Inclusion criteria were first clinic visit of subjects 50 years and older with complaints regarding acquired cognitive decline.

Following screening of the CNIR registry, AS meeting inclusion and exclusion criteria were included in the study. First consecutive JS, attending the clinic at nearest date on the calendar, and meeting inclusion/exclusion criteria were added to the study.

Exclusion criteria included history of traumatic brain injury (TBI) or neurosurgery 1 year preceding the emergence of cognitive deterioration, evaluation for ADHD (attention deficit and hyperactivity disorder), evaluation for insurance purposes and cognitive screening prior to neurosurgical procedures. Patient characteristics, medical background, current diagnoses and follow-up visits were recorded.

Ethnicity was retrieved from the demographical data documentation. When unavailable, patient ethnicity was defined by name. Several studies across the world have examined the issue of ethnicity definition by subject's name. A meta-analysis performed by Pablo Mateos of several studies addressing the issue found a sensitivity of 0.67–0.95 and a specificity of 0.8–1 of such reports (12). As we defined only two broad ethnicities, Arab and Jew, and did not subdivide them according to religion or subethnicity, the specificity and sensitivity are probably much higher.

Ethnic demographic data involving northern Israel residents was extracted from the Central Bureau of Statistics registry.

Cognitive complaints were defined as complaints regarding memory, language, orientation, attention or cognitive slowing. Behavioral complaints were defined when significant changes in comportment and behavior, such as aggression, psychosis, disinhibition, apathy and mood disturbances, were reported. Functional impairment was defined as self-report of any difficulties performing ADL (activities of daily living) or IADL (instrumental ADL).

Dementia, AD and mild cognitive impairment (MCI) were diagnosed according to recommendations from the National Institute on Aging-Alzheimer's Association workgroup (NINCDS-ADRDA criteria) (13, 14). VD (vascular dementia) was diagnosed according to AHA/ASA recommendations (15). Subjects who met both criteria for primary degenerative dementia of the Alzheimer type and neuroimaging features of VD were diagnosed as mixed dementia (MD) (16).

A frontotemporal dementia (FTD) diagnosis was based on clinical presentation combined with ancillary tests (17, 18). Dementia associated with extrapyramidal disease (Lewy body dementia and dementia in Parkinson disease) was diagnosed in patients with prominent extrapyramidal manifestations and a corresponding cognitive profile (19, 20).

Pseudo-dementia was diagnosed in subjects who presented with complaints regarding cognitive deterioration that were clinically judged to be secondary to depression but not to a neurodegenerative condition (21).

Comparison of education, MMSE and duration of symptoms between the two ethnic groups was performed by Mann-Whitney non-parametric test. Patients' age, gender, marital status, medical background, diagnosis in the first and the last follow up, as well as number of follow-ups were compared by Chi square test.

Adjustment of MMSE for age and education years (adjusted MMSE: aMMSE) was based on the MMSE norms recommended by MMSE handbook published by Folstein (22).

*P*-value below 0.005 was considered as statistically significant. Data analysis was performed by SPSS Statistics 25 software.

## Results

**Figure 1:**
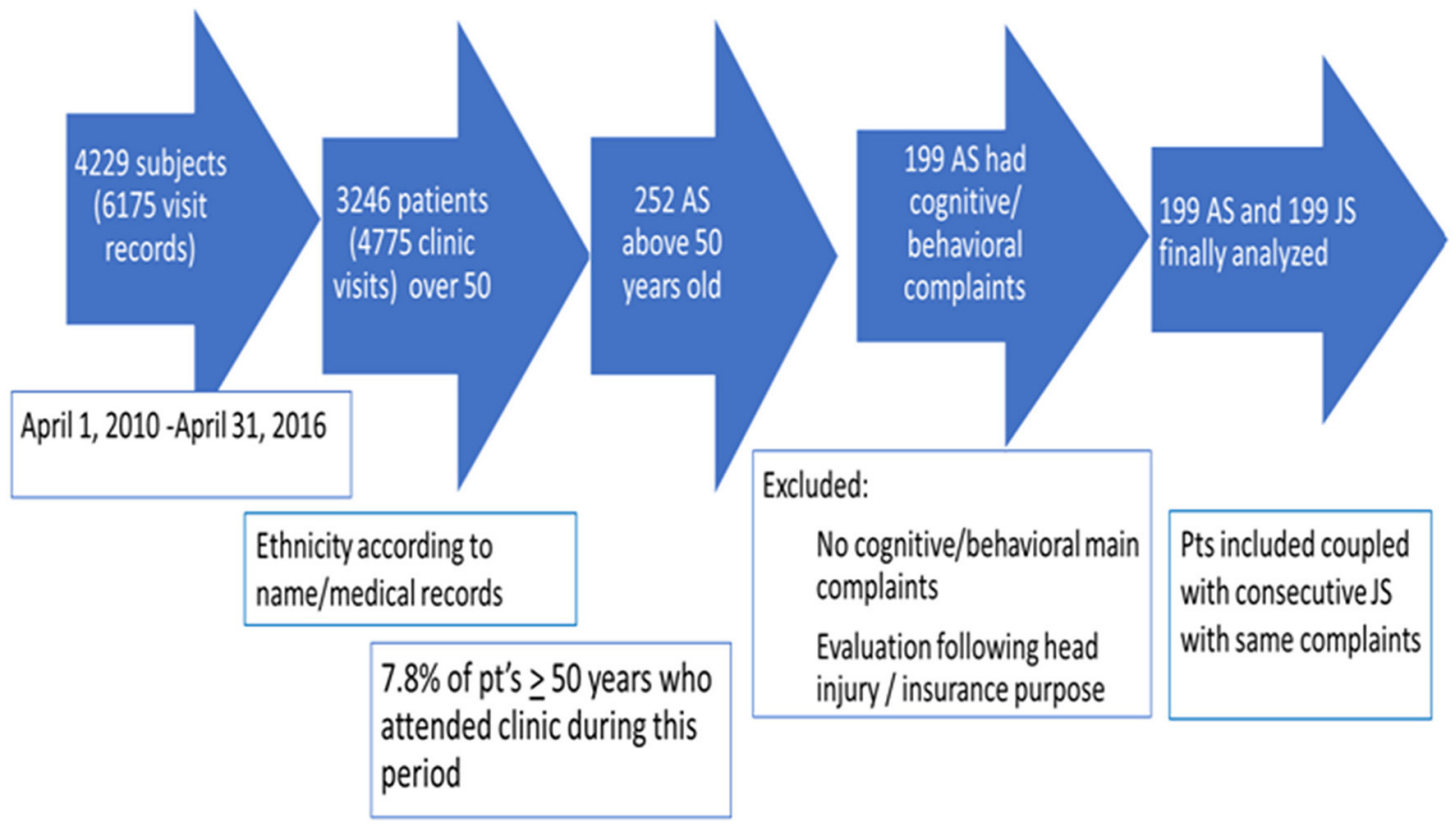
Final enrolment in the study. Pt's, patients.

A total of 6,175 visit records of 4,229 subjects attending the CNIR between April 1, 2010, and April 31, 2016, were reviewed; 3,246 subjects were ≥50 years at the initial visit. Two hundred and fifty-two (7.8%) AS ≥50 years were identified. Fifty-three AS were not included for further analysis due to meeting exclusion criteria or missing data. One hundred and ninety-nine AS were included; in 145 subjects, Arab ethnicity was documented in patients' files, ethnicity of the rest was defined by name. AS were coupled with consecutive JS who met inclusion and exclusion criteria (Figure 1).

Mean age at first visit was 68.4 ± 8.8 for AS and 74.3 ± 9.3 years for JS (*p* < 0.0001). Mean education was 7.7 ± 4.8 years for AS and 11.3 ± 4.8 years for JS (*p* < 0.0001). Seventy-five percent AS and 58% JS were married; 1% AS and 1.5% JS were referred from nursing homes.

Mean duration of cognitive complaints prior to the first visit did not differ between the groups (AS 2.5 ± 3 years, JS 2.4 ± 2.7 years). Initial complaints of both ethnicities included failing memory (97%) and behavioral changes (59%). Functional impairment was reported by 59% of AS and 45% of JS (*p* = 0.005).

MMSE on first evaluation was 19.2 ± 6.9 for AS and 23.1 ± 5.9 for JS; *p* = 0.001 (Table 1). MMSE differences remained significant after correction for age and education years (25.4 ± 22.5 AS, 34.5 ± 22.7 JS, *p* < 0.0001) (22) (adjusted MMSE: aMMSE).

**Table 1:** Demographic data.

**Parameter**	**AS**	**JS**	***P***
	***N* = 199**	***N* = 199**	
Age at first visit (years ± mean)	68.4 ± 8.8	74.3 ± 9.3	<0.0001
Gender: Female, *N* (%)	95 (48)	100 (50)	0.689
Marital status: Married, *N* (%)	150 (75)	116 (58)	<0.0001
Nursing home care, *N* (%)	2 (1)	3 (1.5)	1
Education (years ± mean)	7.7 ± 4.8	11.3 ± 4.8	<0.0001
Family history of memory disorders, *N* (%)	56 (28)	40 (20)	0.391
Duration of symptoms prior to 1st visit (years ± mean)	2.5 ± 3	2.4 ± 2.7	0.618
Complaints on initial presentation			
Cognitive, *N* (%)	196 (98)	190 (95)	0.062
Behavioral, *N* (%)	125 (63)	109 (55)	0.103
Functional, *N* (%)	118 (59)	90 (45)	0.005
MMSE (1st visit)	19.2 ± 6.9	23.1 ± 5.9	<0.0001
Compliance			
Repeated visits: more than one, *N* (%)	90 (45)	96 (48)	0.9
Adherence to prescribed treatment (%)	95	94	1

*AS, Arab subjects; JS, Jew subjects*.

A low aMMSE (<15) was more prevalent in AS (41.2% AS, 25.8% JS, *p* = 0.002), female gender (41.5% female, 25.8% male, *p* = 0.002) and patients with behavioral and functional complaints (43% behavioral, 46% functional complaints; 20% and 19% without behavioral and functional complaints, respectively; *p* < 0.0001). The presence of cognitive complaints was not associated with lower scores on the aMMSE (33% with cognitive complaints, 22% without this complaint had aMMSE below 15, *p* = 0.477).

Past medical diagnoses and prescribed medications as written in the referral are described in Table 2. Vascular risk factors, family history of dementia, history of previous head injury and a background extrapyramidal disease did not differ between the groups. Hypertension diagnosis was more prevalent in JS (58% AS, 69% JS; *p* = 0.017). Background depression (based on medical records) was more prevalent in JS, although not significantly (25% AS, 33% JS; *p* = *0.096*). History of malignancy was more prevalent in the Jewish population (11% AS, 23% JS; *p* = *0.002*). JS used more antidepressants (24% AS, 34% JS; *p* = *0.026*) and thyroid (6% AS, 13% JS; *p* = *0*.026) medications.

**Table 2:** Background diagnoses.

**Parameter**	**Total**		**JS**		**AS**		**Chi-square**
	***N***	**%**	***N***	**%**	***N***	**%**	
Diabetes Mellitus	135	34.0	65	32.7	70	35.2	0.597
Hyperlipidemia	261	65.6	134	67.3	127	63.8	0.527
Hypertension	252	63	138	69	114	58	0.017
Vascular disease^*^	105	26.4	52	26.1	53	26.6	1.000
s/p stroke^**^	100	25.1	54	27.1	46	23.1	0.419
Other intracranial disease^***^	30	7.5	16	8.0	14	7.0	0.85
History of head trauma	39	9.8	23	11.6	16	8.0	0.312
Family history of dementia	96	24.1	40	20.1	56	28.1	0.391
History of malignancy	66	16.6	45	22.6	21	10.6	0.002
Extrapyramidal disease/ Essential tremor	52	13.1	29	14.6	23	11.6	0.457
Recurrent falls	27	6.8	18	9.0	9	4.5	0.109
Vision problems	101	25.4	58	29.1	43	21.6	0.107
Hearing problems	99	24.9	55	27.6	44	22.1	0.246
Sleep apnea	17	4.3	11	5.5	6	3.0	0.322
Depression	114	28.6	65	32.7	49	24.6	0.096
**Medication list**							
Dementia medication	54	13.6	21	10.5	33	16.6	0.081
Cardiovascular medication	331	83.2	171	85.9	160	80.4	0.169
Diabetes	118	29.6	52	26.1	66	33.2	0.124
Antidepressants	115	28.9	68	34.2	47	23.6	0.026
Anticonvulsants including Benzodiazepines	20	5	10	5	10	5	1
Opiates	4	1.0	2	1.0	2	1.0	1
Steroids	5	1.3	3	1.5	2	1.0	1
Antipsychotic	21	5.3	9	4.6	12	6.1	0.511
B12 treatment	65	16.5	28	14.2	37	18.9	0.224
Thyroid medication	38	9.6	26	13.1	12	6.1	0.026

*AS, Arab subjects; JS, Jew subjects*.

* *Heart disease/peripheral vascular disease*.

** *TIA, hemorrhagic and ischemic stroke*.

*** *Benign brain tumor, s/p brain hemorrhage, normal pressure hydrocephalus, white matter disease, s/p encephalitis or meningitis, brain aneurism and AVM*.

Sixty-three AS (32%) and 46 JS (23%) were diagnosed with AD, 29 AS (15%) and 15 JS (8%) were diagnosed with VD. Twenty-three AS (12%) and 41 JS (21%) were diagnosed with MCI. Thirty-seven (19%) AS and 40 (20%) JS were diagnosed with MD. Normal cognition was diagnosed in three AS (2%) and 18 JS (9%). Frontotemporal dementia (FTD) was diagnosed in 0.5% AS and 3% JS. Pseudo-dementia was more prevalent in AS (6.5% AS; 0.5% JS). Dementia associated with extrapyramidal disease was diagnosed in 5 AS and 4 JS. The diagnosis could not be established at the initial visit in 11%. These subjects were referred for further evaluations.

Following initial recommendations, 90 (45%) AS and 96 (48%) JS returned to at least one follow-up visit; 25% AS and 25% JS returned to recurrent (≥2) follow-up visits; mean number of follow up visits was 1.15 ± 1.8 for AS and 1.2 ± 2 for JS, *p* = 0.9. Two months to 10 years elapsed between the first to last visit; mean follow up was 2.58 ± 1.7 years for AS and 2.88 ± 2.38 years for JS; *p* = 1. Ninety-five percent of subjects followed the prescribed treatment.

During the last follow-up visit, normal cognition was recorded in four (4%) JS and no AS. Fifty-three AS (59%) and 39 JS (41%) were diagnosed with AD, three AS (3%) and 15 (16%) JS were diagnosed with MCI, 3% AS and 3% JS were found to have VD. Similar rates of MD were found in both groups; 19% AS and 23% JS (Table 3). Diagnosis was still deferred in 3% of subjects. Compared to the first visit, patients had similar rates of MD and two times higher rates of AD in both ethnicities. Normal cognition, MCI and VD were less prevalent on the last visits.

**Table 3:** Diagnosis in the first/last visit.

		**Total**		**JS**		**AS**		***p*-value**
		***N***	**%**	***N***	**%**	***N***	**%**	
**First visit**	All patients	398		199		199		0.000
	Normal cognition	21	5%	18	9%	3	2%	
	MCI	64	16%	41	21%	23	12%	
	Alzheimer dementia	109	27%	46	23%	63	32%	
	Vascular dementia	44	11%	15	8%	29	15%	
	Mixed dementia	77	19%	40	20%	37	19%	
	Frontotemporal dementia	7	2%	6	3%	1	0.5%	
	Extrapyramidal disease	9	2%	4	2%	5	2.5%	
	Pseudo-dementia	14	3.5%	1	0.5%	13	6.5%	
	Other	10	2.5%	5	2.5%	5	2.5%	
	UNKNOWN	43	11%	23	12%	20	10%	
**Last visit**	All patients	186		96		90		0.000
	Normal cognition	4	2%	4	4%	0	0%	
	MCI	18	10%	15	16%	3	3%	
	Alzheimer dementia	92	50%	39	41%	53	59%	
	Vascular dementia	6	3%	3	3%	3	3%	
	Mixed dementia	39	21%	22	23%	17	19%	
	Frontotemporal dementia	5	3%	5	5%	0	0%	
	Extrapyramidal disease	6	3%	4	4%	2	2%	
	Pseudo-dementia	5	3%	0	0%	5	6%	
	Other	4	2%	2	2%	2	2%	
	UNKNOWN	5	3%	1	1%	4	4%	

*AS, Arab subjects; JS, Jew subjects*.

## Discussion

Despite the fact that the Arab population constitutes 37% of northern Israel residents 55 years and older and 17% of the Haifa district residents (23), AS constituted only 7.8% of the subjects that were evaluated in the CNIR.

In Israel, evaluations for cognitive deterioration within tertiary dementia clinics are only partially reimbursed by the national health care providers. According to the Israeli bureau of statistics, Arab salaried employees earn about 65% less than their Jewish counterparts (11). Accordingly, Arab households spend 42% less money on supplementary healthcare insurance than do Jewish ones (24). Indeed, prior studies support a correlation between lower income and self-reported health in the Israeli Arab population (25). Therefore, the poorer financial status of AS may partially account for their underrepresentation in the clinic.

An additional cause for the under-representation of AS may be ethnicity dependent beliefs regarding dementia. AD may be associated with futility and stigma regarding “mental diseases and dementia” which may impede help seeking (26). Previous research exploring stigma among caregivers of persons with AD in Israel report pronounced stigma in Arab compared to Jewish population and suggest that a lower level of education may account for this difference (27). Also, prior works suggest that ethnic minorities often perceive cognitive decline as part of normal aging, delaying seeking medical help (28).

A low referral rate of ethnic minorities to cognitive clinics was shown in previous works (29); this may be another reason for AS underrepresentation and stand as an important intervention point.

To adjust for the imbalance in the number of subjects attending our clinic, we evaluated AS and immediately consecutive JS dyads, as stated. AS differed significantly from JS with regard to demographic and clinical characteristics.

AS were younger at their first visit compared to JS (68.4 ± 8.8 vs. 74.3 ± 9.3; *p* < 0.0001), nevertheless, they were referred for evaluation when they already reported functionally impairment and had a lower MMSE (Table 1). Similar findings were reported in Asian and black minorities residing in London, UK (30) and in the Latino minority in Philadelphia (31).

AS had a higher rate of AD (32 vs. 23% in JS) and of VaD (15 vs. 8% in JS). JS were more frequently diagnosed with normal cognition (2% in AS; 9% in JS) and with MCI (12% in AS; 21% in JS). This data correlates with previous studies that show higher rates of AD and VaD in the Arab population aged ≥60 (5, 6, 32).

A systematic review and meta- analysis found that, worldwide, people from minority ethnic groups with dementia access healthcare services in later stages of their illness (33). Our study supports this finding. Although our study did not show any differences in the duration of the reported symptoms between the onset of cognitive complaints and the first CNIR visit (2.5 ± 3 in AS, 2.4 ± 2.7 in JS), this value may be under-reported. This is supported by low rates of MCI, lower MMSE and higher rates of functional impairment documented on the first visit in AS in our study.

Other reasons for similar duration of symptoms despite poorer cognitive performance may be lower basic MMSE and a higher pace of cognitive decline in AS populations.

Higher AD prevalence and younger age at presentation of AS may have several reasons.

Alzheimer's disease is frequently heritable (34) and high rates of consanguineous marriages are reported between Israel Arabs (9, 35).

Compared to JS, AS had fewer schooling years (Table 1). Lower formal education in the Arab population in Israel was reported previously and is thought to reflect socio-demographic and ethno-religious elements (11, 36). Lower mean years of formal education tend to characterize members of minority groups compared to the majority group and were associated with AD in previous studies (37) and may partially explain higher AD rates in Arab population (8).

Vascular risk factors, such as diabetes and hypertension, are commonly associated with an increased risk for AD and dementia (38, 39). In the present study, the prevalence of diabetes was similar between AS and JS (Table 2), but, according to previous work, diabetes prevalence is higher in the Arab population (40). Similarly, hypertension was less prevalent in AS (Table 2), although poor blood pressure control was previously reported in the Arab population in Israel (41).

Depression is also regarded as a risk factor for AD (42, 43). Kaplan et al. (44) found depression rates 2.5 times higher in the Arab population compared to the Jewish population in Israel. In the present study, clinical, co-morbid diagnosis of depression was similar in both populations, while antidepressant use was higher in JS. As we collected community diagnoses, a possible reason for the discrepancy between our data and previous data is under-diagnosis and/or under-treatment of depression in ethnic minorities (45–47).

Interestingly, pseudo-dementia was diagnosed far more commonly in AS (13 vs. 1). Eleven of those AS patients had a previous depression diagnosis and nine were treated medically for depression. Somatization disorders in general are more frequently found in the Arab society (48, 49) and due to cultural and religious elements depression particularly tends to present with somatic complaints in Arab society (50, 51).

The prevalence of dementia in our study was similar to prior works. First visit diagnosis was AD in 27% of subjects, VaD in 11% and MD in 19%. Previous pathological and clinical studies in elderly onset dementia report AD in 20–40% of patients (52–54), VaD in 7.5–24% (53, 54), and MD in 13–40% (16, 54). During follow-up visits AD diagnosis was more frequent than in the first visit (50%), probably as MCI patients converted to AD and patients with deferred diagnosis were diagnosed with AD.

Our study showed a higher rate of past malignancy diagnosis in JS (21 AS, 45 JS). A higher rate of malignancy in the Jewish compared to the Arab population is consistent with the Israel cancer register (55) and may be due to differences in diet, genetic factors, the lower mean age of the Arab population and the more prevalent urban way of life of the Jewish population (56).

A low aMMSE (<15) was more prevalent in patients with behavioral and functional complaints. The presence of cognitive complaints was not associated with lower scores on the aMMSE. Prior studies did not show any difference in the number of cognitive complaints between MCI and dementia patients (57). Memory complaints were more common in mild-moderate dementia than in severe dementia (58). The presence of cognitive complaints usually does not correlate with dementia severity as those complaints usually evolve in the early stages of MCI and dementia and are the basic criteria for MCI and dementia diagnosis. As dementia progresses it usually affects more behavioral aspects (59) and functional abilities, correlating in our study with low aMMSE.

Although Arab patients are underrepresented in our study and may arrive at the tertiary clinic with more advanced disease, they have the same rate of compliance with treatment and continue follow up as the Jewish population. This good compliance with treatment and follow-up may reinforce the importance of creating public policies to bring Arab patients to the clinics earlier.

To the best of our knowledge, our study is the first that directly compares prevalence and characteristics of dementia in the two main ethnic groups in Israel (8). Its limitation is that it is retrospective and descriptive in nature, raising the need for future prospective comparative research.

In summary, our study reveals that AS arrive for evaluation younger but already report functional impairment and have lower MMSE, suggesting that AS under-representation in the clinic most probably reflects lower usage of this medical service. Reasons can be poorer awareness of dementia and lower referral rate, lower socioeconomic status and educational level and poorer control of risk factors in this population.

The importance of this study is in planning preventive measures and future interventions in order to reduce the disparity between ethnicities. Proactive educational interventions in the Arab community along with raising awareness about the importance of memory clinics among family physicians, district neurologists and the population itself, should be considered.

## Data Availability Statement

The datasets presented in this article are not readily available because the data has been sufficiently de-identified and is available upon request. Requests to access the datasets should be directed to the corresponding author.

## Ethics Statement

The studies involving human participants were reviewed and approved by Institutional Review Boards of Rambam Health Care Campus (# 0680-19-RMB). Written informed consent from the participants was not required to participate in this study in accordance with the national legislation and the institutional requirements.

## Author Contributions

PS and JA conceived the research idea. PS, RB, NY, and JA performed evaluations of the subjects in the Rambam cognitive clinic. PS collected retrospect information about the clinic visits, prior diagnosis, treatment and follow ups and wrote the manuscript in consultation with JA and TF. All authors contributed to the article and approved the submitted version.

## Conflict of Interest

The authors declare that the research was conducted in the absence of any commercial or financial relationships that could be construed as a potential conflict of interest.
